# Hydrogen sulfide alleviates hyperoxia effects on mitochondria in human developing airway smooth muscle

**DOI:** 10.1172/jci.insight.191475

**Published:** 2026-03-10

**Authors:** Colleen M. Bartman, Michael Thompson, Samantha K. Hamrick, Niyati A. Borkar, Daniel Pfeffer-Kleemann, Preetham Ravi, Marta Schiliro, Yak Nak, Christian Vivar Ramon, Li Drake, Y.S. Prakash, Christina Pabelick

**Affiliations:** 1 Department of Anesthesiology and Perioperative Medicine, Mayo Clinic, Rochester, Minnesota, USA.; 2 Department of Anesthesiology and Critical Care Medicine, School of Medicine and Surgery, University of Milano-Bicocca, Monza, Italy.; 3 University of Missouri School of Medicine, Columbia, Missouri, USA.; 4 University of Pittsburgh School of Medicine, Pittsburgh, Pennsylvania, USA.; 5 Department of Physiology and Biomedical Engineering, Mayo Clinic, Rochester, Minnesota, USA.

**Keywords:** Cell biology, Pulmonology, Therapeutics, Asthma, Calcium signaling, Mitochondria

## Abstract

Moderate hyperoxia (30%–60% O_2_) in premature infants promotes bronchial airway hyperresponsiveness (AHR) via airway smooth muscle (ASM), a key regulator of bronchoconstriction, bronchodilation, and remodeling. Understanding how O_2_ exposure drives long-term bronchial changes in prematurity is critical for developing therapies for airway disease across the lifespan. Premature lungs have immature antioxidant defenses, potentially due to disrupted mitochondrial dynamics, increasing susceptibility to O_2_-induced oxidative stress. Thus, mitochondrial homeostasis is highly relevant to ASM dysfunction and airway disease. We propose that hyperoxia in prematurity promotes mitochondrial dysfunction, and that the gasotransmitter hydrogen sulfide (H_2_S) mitigates O_2_-induced mitochondrial damage in developing ASM. Human fetal ASM (fASM) cells were exposed to moderate hyperoxia to investigate the effects of exogenous H_2_S donors (GYY4137, AP39) and stabilization of cystathionine β-synthase (CBS), an H_2_S biosynthetic enzyme, on mitochondrial structure and function. Hyperoxia impaired fASM cell mitochondrial integrity, while H_2_S donors in particular, or CBS stabilization attenuated adverse O_2_ effects on mitochondrial morphology, ROS, respiration, calcium regulation, and contractility. These findings highlight the therapeutic potential of H_2_S in the premature lung exposed to moderate hyperoxia.

## Abstract 2

These studies investigate the alleviating role of hydrogen sulfide in mitochondrial homeostasis of developing human airway cells exposed to moderate hyperoxia.

## Graphical abstract

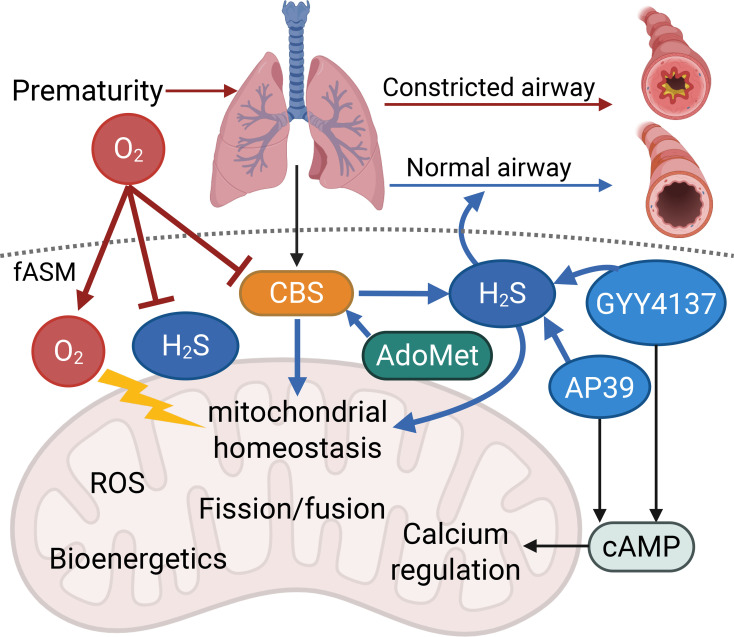

## Introduction

Moderate hyperoxia (30%–60% O_2_) is a common and necessary therapy following premature birth (<34 weeks gestation) to help underdeveloped lungs maintain oxygenation and ventilation. Clinical practice favors moderate hyperoxia given the well-documented deleterious effects of high O_2_ (80%–90%), which is classically associated with bronchopulmonary dysplasia (1–3). However, even moderate hyperoxia poses risks by promoting bronchial airway hyperresponsiveness (AHR) via effects on airway smooth muscle (ASM). ASM contributes to impaired bronchodilation and airway remodeling, particularly in developing airways (4–8). Premature infants are highly susceptible to effects of O_2_ because early transition to ex utero life requires rapid adjustment from a relatively hypoxic to hyperoxic environment. This places added stress on the already immature lungs, further exacerbated by supplemental O_2_. Since antioxidant systems mature later in gestation in fetal lungs (9–11), the preterm lung is ill-equipped to manage increased O_2_. Thus, premature birth and early exposures to even moderate O_2_ levels can have deleterious lifelong impacts on airway structure and function (12–16) and contribute to pediatric asthma (12, 17–19) involving chronic inflammation, AHR, and airway remodeling. Understanding mechanisms by which moderate O_2_ initiates and sustains long-term bronchial airway changes during the perinatal period is important for identifying targeted therapies for pediatric airway disease.

We propose that the gasotransmitter hydrogen sulfide (H_2_S) attenuates O_2_-induced mitochondrial dysfunction in developing human fetal ASM (fASM). Previous work from our group demonstrated the relevance of H_2_S and its downstream effects using both an in vitro fASM model (20) and an in vivo neonatal mouse model of moderate hyperoxia (21). Expression of a key H_2_S synthesis enzyme, cystathionine β-synthase (CBS), is reduced by O_2_ exposure in fASM (20). Inhibiting endogenous H_2_S increases cytoplasmic calcium responses to bronchoconstrictors, whereas exogenous H_2_S donors sodium hydrosulfide (NaHS, acute) and GYY4137 (chronic) attenuate these effects (20). In neonatal mice, moderate hyperoxia (40%–50% O_2_) increases ASM thickness, collagen deposition, and AHR (22, 23), while the long-acting H_2_S donor GYY4137 mitigates these deleterious effects of O_2_ on developing airway structure and function (21).

H_2_S may exert beneficial effects in moderate hyperoxia by regulating mitochondrial homeostasis and intracellular calcium. Since H_2_S is catabolized via mitochondrial enzymes, its production and function are closely tied to mitochondrial health. Mitochondrial morphology is dynamically regulated via fission and fusion, matching mitochondrial structure to metabolic demand and O_2_ consumption (24, 25). Within mitochondria, H_2_S increases glutathione (GSH) production to protect against oxidative stress (26), acting as a cofactor to eliminate free radicals or detoxify products affected by high levels of ROS (27), and inhibits inflammation via Nrf2 and NF-κB regulation (28–30). H_2_S may exert its effects via cAMP, increasing cAMP and protein kinase A (PKA) in neuronal, epithelial, and smooth muscle cells while attenuating effects of β-2 adrenoceptor (β2-AR) in the vasculature (31, 32). Mitochondrial dynamics, respiration, and calcium regulation are also central to ASM function and airway disease, particularly in the context of O_2_ and prematurity where cells unable to adapt to higher O_2_ are at risk for mitochondrial dysfunction. Disrupted mitochondrial bioenergetics and altered mitochondria–sarcoplasmic reticulum (SR) calcium exchange impair cellular contractility and contribute to AHR (33, 34).

In this study, we link H_2_S signaling via CBS to mitochondrial homeostasis through the cAMP pathway in fASM exposed to moderate O_2_. We hypothesize that moderate hyperoxia blunts H_2_S availability and promotes mitochondrial dysfunction, while exogenous H_2_S counteracts O_2_ effects. Using fASM cells with CBS knockdown (KD), CBS stabilization (*S*-adenosylmethionine; AdoMet), or treatment with general or mitochondrial-specific H_2_S donors (GYY4137 vs. AP39), we demonstrate that moderate hyperoxia disrupts mitochondrial morphology, calcium ([Ca^2+^]_m_), ROS production and respiration, as well as cytoplasmic calcium ([Ca^2+^]_c_) and contractility. Such effects were alleviated by H_2_S modulation via cAMP signaling, highlighting a potential therapeutic strategy to counteract hyperoxia effects on bronchoconstriction in developing airways.

## Results

### Generation of CBS stable KD in fASM cells using lentiviral particles.

**Figure 1:**
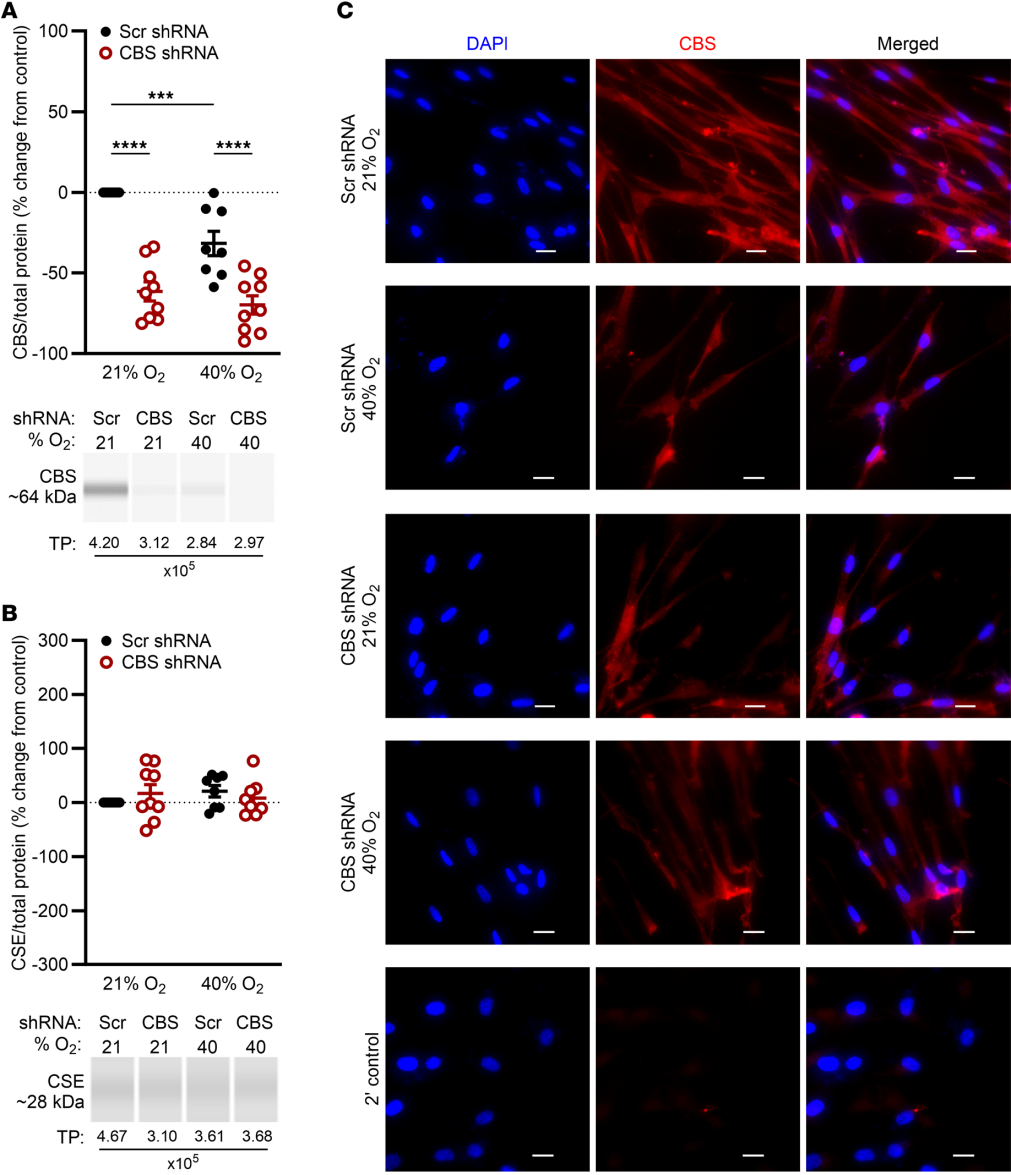
Generation of CBS stable KD in fASM cells using lentiviral particles. Lentiviral shRNA particles were used to generate CBS-KD and Scr control fASM cells in 9 patient lines. Protein expression was quantified by Jess, an automated and quantitative immuno-capillary system that generates a digital Western blot (representative images shown). Protein expression was normalized to total protein (TP) chemiluminescence (total protein values indicated below representative images). Graphs represent percentage change from each fASM cell line’s respective Scr control in 21% O_2_. (**A**) Validation of CBS-KD fASM cell lines in 21% and 40% O_2_ compared to Scr control. CBS peaks were identified using approximately 64 kDa as the molecular weight. (**B**) CSE protein expression in CBS-KD and Scr fASM cells in 21% and 40% O_2_. CSE peaks were identified using approximately 28 kDa as the molecular weight. ***P < 0.001; ****P < 0.0001 by 2-way ANOVA with Bonferroni’s correction for multiple comparisons. Data are represented as mean ± SEM; *n* = 9 fASM lines/group. (**C**) Representative images are from 1 of 5 immunofluorescence experiments of CBS protein expression in CBS-KD and Scr fASM cells exposed to 21% or 40% O_2_ for 48 hours. Appropriate secondary control for immunofluorescence microscopy is shown. Red, CBS; blue, DAPI. Scale bars: 20 μm.

Our previous work identified the H_2_S-producing enzyme CBS as a key player in fASM where O_2_ exposure reduced its expression (20). To investigate the role of CBS in fASM, lentiviral shRNA KD cell lines were generated and validated by capillary immunoassay or immunofluorescence. In 21% O_2_, CBS-KD fASM cells showed decreased CBS protein expression (~34%–82% KD) compared with scrambled (Scr) controls. In Scr fASM cells, CBS protein expression was lower in 40% O_2_ than 21% O_2_. In 40% O_2_, CBS-KD fASM cells showed lower CBS expression (~46%–93% KD) compared with Scr fASM cells exposed to either 21% or 40% O_2_ (Figure 1A). No compensatory effects were noted across groups in expression of cystathionine γ-lyase (CSE), another enzyme involved in generating H_2_S (Figure 1B). Representative digital blots are shown. Immunofluorescence confirmed reduced CBS expression in Scr fASM cells exposed to 40% O_2_ and in CBS-KD fASM cells under both 21% and 40% O_2_ conditions (Figure 1C; representative images).

### CBS KD alters mitochondrial morphology and ROS in fASM cells exposed to moderate hyperoxia.

**Figure 2:**
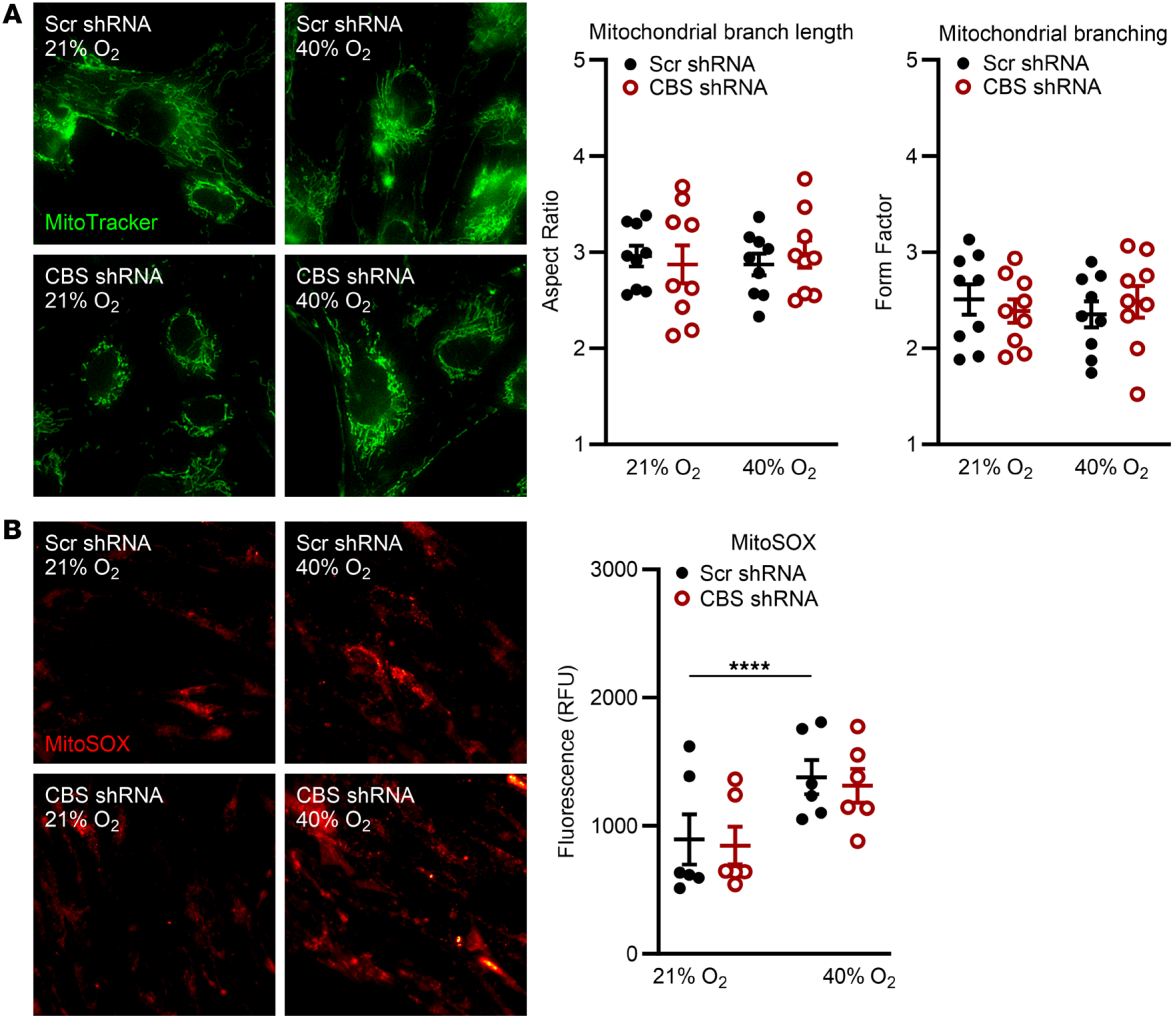
CBS KD alters mitochondrial morphology and ROS in fASM cells exposed to moderate hyperoxia. CBS-KD and Scr control fASM cells exposed to 21% or 40% O_2_ for 48 hours were stained with (**A**) MitoTracker Green FM (mitochondrial morphology marker) and (**B**) MitoSOX (mitochondrial superoxide indicator). (**A**) Representative fluorescence images are 1 of 5 experiments with MitoTracker-dyed CBS-KD and Scr shRNA fASM cells in 21% and 40% O_2_. Mitochondrial branch length (aspect ratio) and mitochondrial branching (form factor) were quantified using the MitoMorph macro and ImageJ software. (**B**) Representative fluorescence images are 1 of 6 experiments with MitoSOX-dyed CBS-KD and Scr shRNA fASM cells in 21% and 40% O_2_. MitoSOX fluorescence (RFU) was quantified. ****P < 0.0001 by 2-way ANOVA with Bonferroni’s correction for multiple comparisons. Data are represented as mean ± SEM; *n* = 6–9 fASM lines/group.

Given involvement of CBS in the transsulfuration pathway, we assessed its role in mitochondrial regulation through either direct or indirect pathways. MitoTracker imaging of morphology showed no differences in mitochondrial branch length (aspect ratio) or branching (form factor) in Scr fASM cells exposed to 40% versus 21% O_2_ for 48 hours (Figure 2A; representative images). Analysis of MitoSOX (mitochondrial superoxide indicator) staining revealed increased mitochondrial ROS in Scr fASM cells exposed to 40% O_2_ versus 21% O_2_ at 48 hours. CBS-KD fASM cells also showed increased ROS in 40% O_2_ but did not exacerbate hyperoxia-induced mitochondrial ROS production compared to Scr controls (Figure 2B; representative images).

### CBS KD increases [Ca^2+^]_m_ responses, with variable effects on mitochondrial respiration.

**Figure 3:**
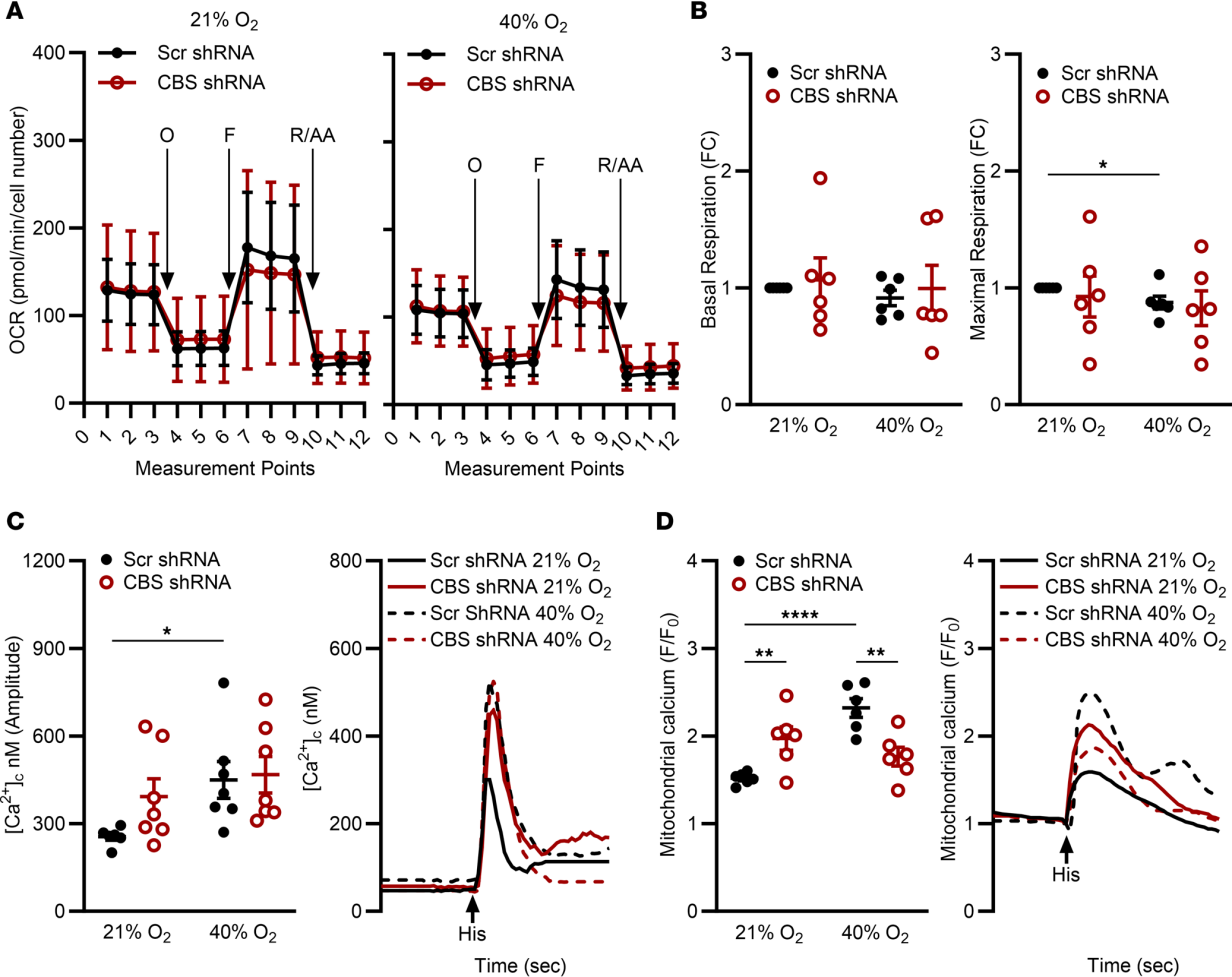
CBS KD increases [Ca^2+^]_m_ responses to histamine, with variable effects on mitochondrial respiration. (**A** and **B**) Scr shRNA and CBS-KD fASM cells were exposed to 21% or 40% O_2_ prior to Seahorse mitochondrial stress test. Values normalized to cell number. O, oligomycin; F, FCCP = carbonyl cyanide-4-(trifluoromethoxy)phenylhydrazone; R/AA, rotenone/antimycin A. (**A**) Time course of mitochondrial stress tests. (**B**) Basal respiration and maximal respiration. Data are represented as fold change from each fASM line control (Scr shRNA in 21% O_2_). *P < 0.05 by unpaired *t* test. *n* = 6 fASM lines/group. (**C**) CBS-KD and Scr fASM cells were exposed to 21% or 40% O_2_ for 48 hours prior to live cell fluorescent imaging of [Ca^2+^]_c_ response to 10 µm histamine (Fura-2/AM). Amplitude was calculated from baseline to maximum peak [Ca^2+^]_c_ response. (**D**) CBS-KD and Scr fASM cells were exposed to 21% or 40% O_2_ for 48 hours prior to live cell fluorescent imaging of [Ca^2+^]_m_ response to 10 µm histamine (Rhod-2). [Ca^2+^]_m_ response was calculated between the background-adjusted baseline (background fluorescence subtraction) to maximum peak [Ca^2+^]_m_ response (F/F_0_). In **C** and **D**, representative tracings are shown (1 from 6–7 fASM cell lines). *P < 0.05; **P < 0.01; ****P < 0.0001 by 2-way ANOVA with Bonferroni’s correction for multiple comparisons (**C** and **D**). Data are represented as mean ± SEM; *n* = 6–7 fASM lines/group.

Using a Seahorse Bioanalyzer, we previously showed that O_2_ influences mitochondrial respiration (35). Here, Scr and CBS-KD fASM cells were exposed to 21% or 40% O_2_ for 24–48 hours before a Seahorse mitochondrial stress test (Figure 3A). CBS KD had variable effects on basal respiration, independent of O_2_. Maximal respiration was reduced in Scr fASM cells exposed to 40% O_2_ with no additional effect of CBS KD (Figure 3B).

We previously showed that 40% O_2_ exposure increases [Ca^2+^]_c_ response of fASM to bronchoconstrictors such as histamine (20, 36, 37). Calcium imaging showed increased [Ca^2+^]_c_ in Scr fASM cells exposed to 40% O_2_. With 21% O_2_, CBS KD resulted in marked increase in [Ca^2+^]_c_ response trending toward significance compared with Scr fASM. In 40% O_2_, CBS-KD fASM cells also showed increased [Ca^2+^]_c_ response to histamine, but not different from Scr fASM cells in 40% O_2_. The hyperoxia effect on [Ca^2+^]_c_ was thus not exacerbated by CBS KD (Figure 3C; representative images).

[Ca^2+^]_m_ imaging showed increased responses in Scr fASM cells exposed to 40% O_2_. In 21% O_2_, CBS KD increased [Ca^2+^]_m_ response compared with Scr fASM cells but interestingly, CBS KD decreased [Ca^2+^]_m_ in 40% O_2_ compared with Scr controls (Figure 3D; representative tracings).

### CBS stabilization attenuates O_2_ effects on calcium signaling.

**Figure 4:**
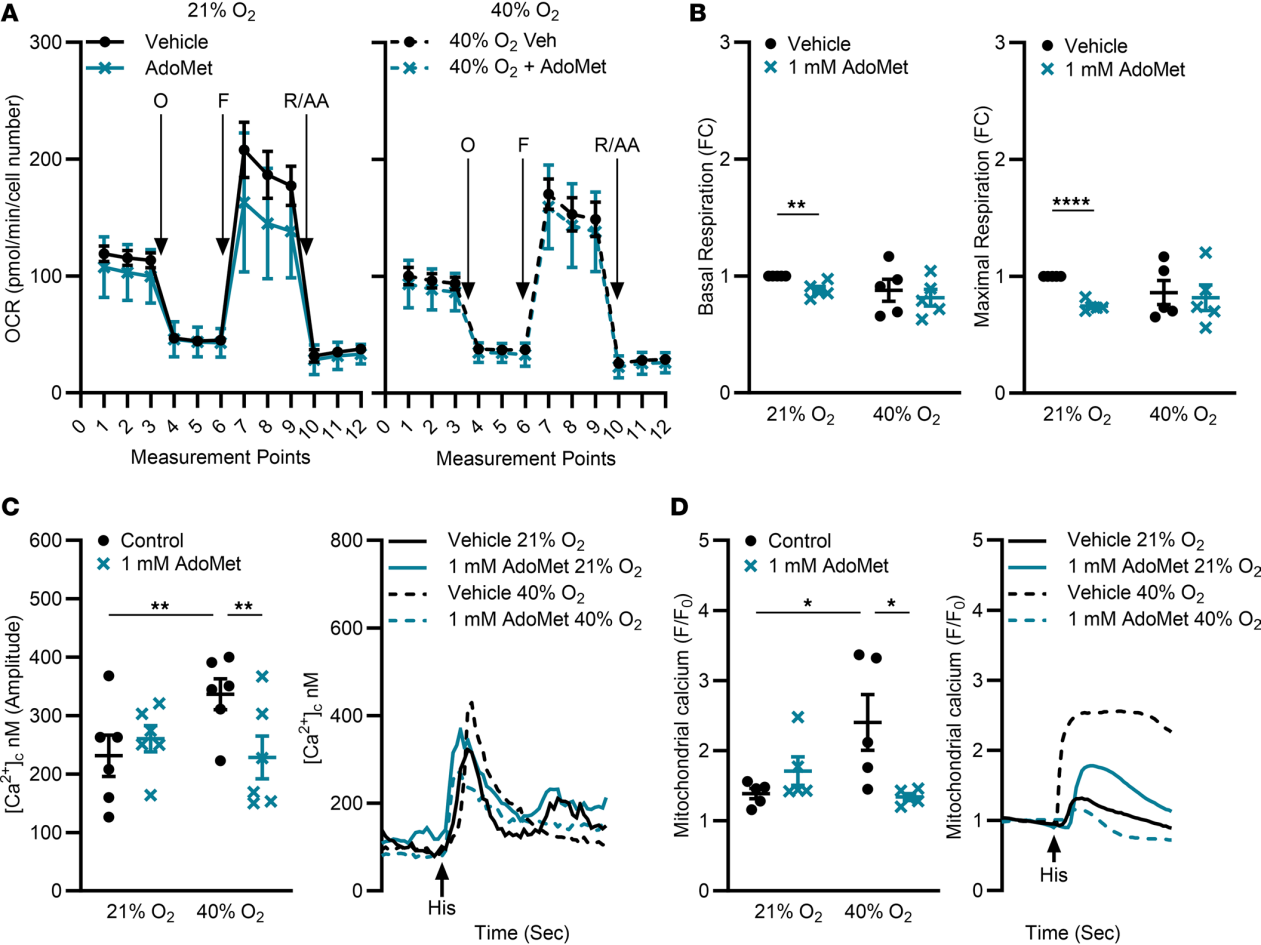
CBS stabilization via AdoMet attenuates effects of O_2_ on calcium signaling. (**A**) Untransduced fASM cells were exposed to 21% or 40% O_2_ prior to mitochondrial stress test using a Seahorse Bioanalyzer as described in Figure 3A. Time courses of mitochondrial stress tests. O, oligomycin; F, FCCP = carbonyl cyanide-4-(trifluoromethoxy)phenylhydrazone; R/AA, rotenone/antimycin A. (**B**) Basal respiration and maximal respiration were calculated. Data are represented as fold change from each fASM line control (vehicle in 21% O_2_). **P < 0.01; ****P < 0.0001 by unpaired *t* test. Data are represented as mean ± SEM; *n* = 5 fASM lines/group. (**C**) Untransduced fASM cells were treated with 1 mM AdoMet or vehicle control and exposed to 21% or 40% O_2_ for 48 hours prior to live cell fluorescent imaging of [Ca^2+^]_c_ response to 10 µm histamine (Fura-2/AM). (**D**) Untransduced fASM cells were treated with 1 mM AdoMet or vehicle control and exposed to 21% or 40% O_2_ for 48 hours prior to live cell fluorescent imaging of [Ca^2+^]_m_ response to 10 µm histamine (Rhod-2) and response was calculated between the background-adjusted baseline (background fluorescence subtraction) to maximum peak [Ca^2+^]_m_ response (F/F_0_). In **C** and **D**, representative tracings are shown (1 from 5–6 fASM lines). *P < 0.05; **P < 0.01 by 2-way ANOVA with Bonferroni’s correction for multiple comparisons (**C** and **D**). Data are represented as mean ± SEM; *n* = 5–6 fASM lines/group.

To investigate the effect of CBS stabilization, untransduced fASM cells were treated with vehicle control or 1 mM AdoMet, an allosteric CBS activator that stabilizes it against degradation (38, 39). Samples then underwent Seahorse mitochondrial stress tests (Figure 4A). AdoMet decreased both basal and maximal respiration in 21% O_2_ (Figure 4B).

AdoMet had minimal effects on [Ca^2+^]_c_ in 21% O_2_ but attenuated 40% O_2_–induced increases in both [Ca^2+^]_c_ and [Ca^2+^]_m_, restoring responses to levels comparable to 21% O_2_ controls (Figure 4, C and D; representative tracings). These results demonstrated an attenuating effect of CBS stabilization in fASM cells exposed to moderate hyperoxia.

### Exogenous H_2_S donors attenuate O_2_-induced mitochondrial dysfunction.

**Figure 5:**
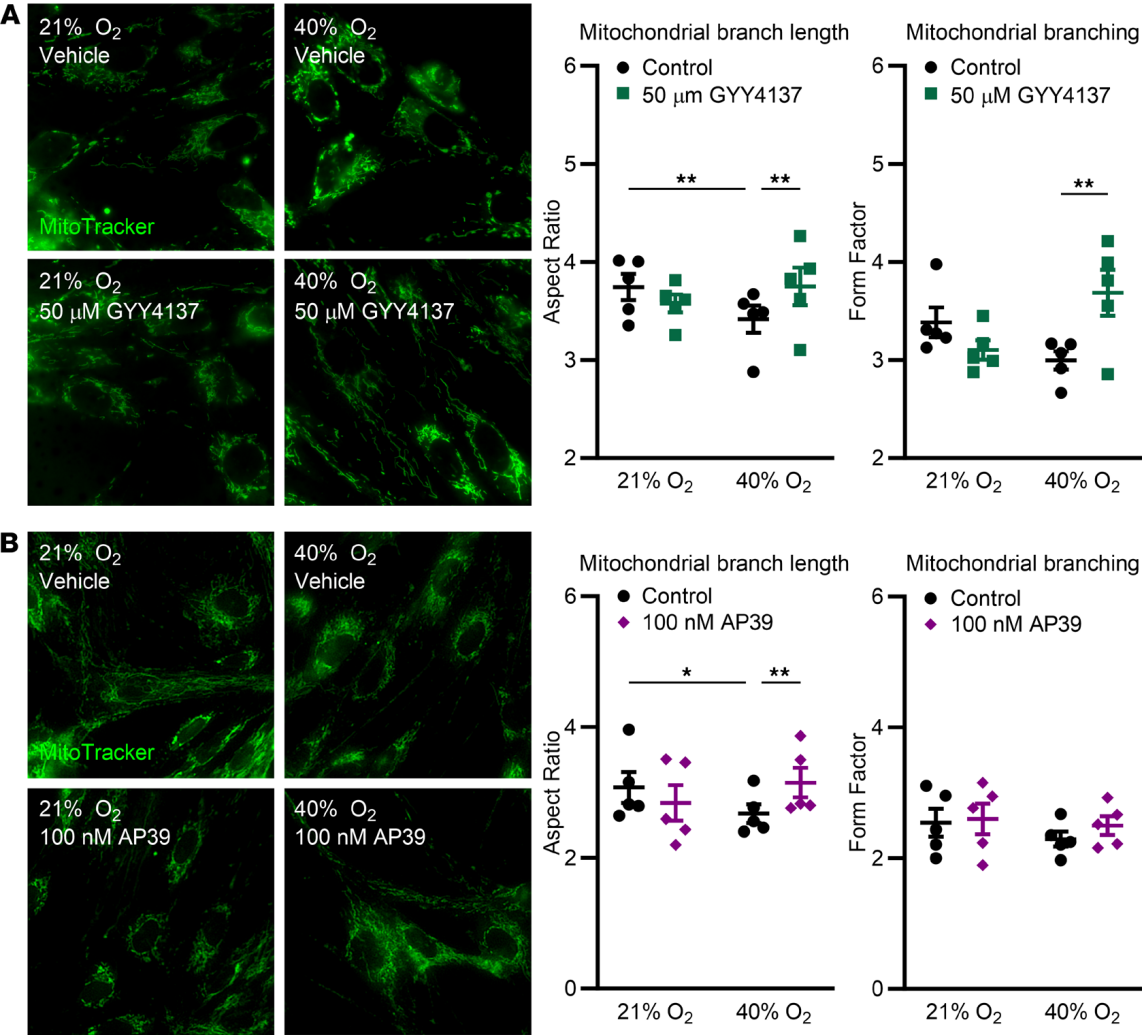
Exogenous H_2_S donors attenuate O_2_-induced mitochondrial dysfunction. fASM cells were treated with (**A**) 50 µm GYY4137 (slow-releasing H_2_S donor) or (**B**) 100 nM AP39 (mitochondrial-targeting H_2_S donor) and exposed to 21% or 40% O_2_ for 48 hours. Magnification: 40x objective, 400x overall. MitoTracker Green was used to quantify mitochondrial branch length (aspect ratio) and mitochondrial branching (form factor) using the MitoMorph macro and ImageJ software. Representative fluorescence images shown are from 1 of 5 fASM lines used in each experiment. *P < 0.05; **P < 0.01 by 2-way ANOVA with Bonferroni’s correction for multiple comparisons. Data are represented as mean ± SEM; *n* = 5 fASM lines/group.

To evaluate effects of O_2_ and exogenous H_2_S donors on mitochondrial morphology, fASM cells were treated with 50 µm GYY4137 (general H_2_S donor), 100 nM AP39 (mitochondria targeted donor), or vehicle (control) and exposed to 21% or 40% O_2_ for 48 hours, followed by MitoTracker Green staining. In vehicle controls, mitochondrial branch length (aspect ratio) decreased in 40% O_2_ while branching (form factor) was markedly decreased in 40% O_2_. Both effects were attenuated by GYY4137 (Figure 5A; representative MitoTracker images). In comparison, AP39 selectively restored hyperoxia effects on mitochondrial branch length but not branching (Figure 5B; representative MitoTracker images).

### Exogenous H_2_S donors attenuate O_2_ effects on mitochondrial ROS and improve respiration.

**Figure 6:**
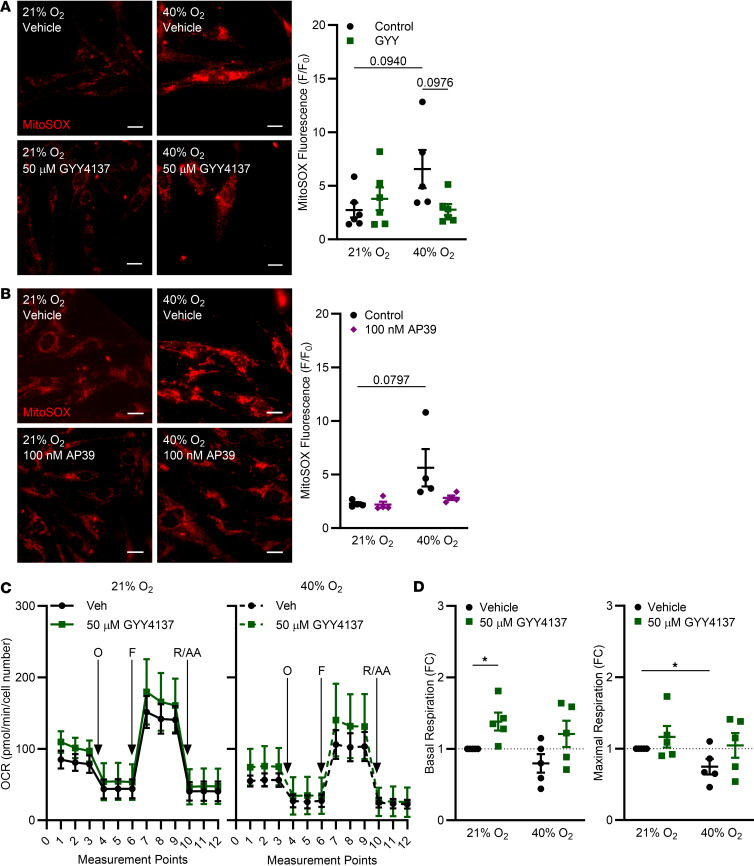
Exogenous H_2_S donors attenuate O_2_ effects on mitochondrial ROS and improve respiration. fASM cells were treated with (**A**) 50 µm GYY4137 (slow-releasing H_2_S donor) or (**B**) 100 nM AP39 (mitochondrial-targeting H_2_S donor) and exposed to 21% or 40% O_2_ for 48 hours. Cells were stained with MitoSOX and fluorescence (F/F_0_) quantified. (**A** and **B**) Representative fluorescence images shown are from one of 4 or 6 fASM used in each experiment. Scale bars: 20 μm. Significance by 2-way ANOVA with Bonferroni’s correction is indicated on the graphs. Data are represented as mean ± SEM; *n* = 4–6 fASM lines/group. (**C**) fASM cells were treated with 50 µm GYY4137 and exposed to 21% or 40% O_2_ for 48 hours prior to mitochondrial stress test using a Seahorse Bioanalyzer. Time courses of mitochondrial stress tests are shown. O, oligomycin; F, FCCP = carbonyl cyanide-4-(trifluoromethoxy)phenylhydrazone; R/AA, rotenone/antimycin A. (**D**) Basal respiration and maximal respiration were calculated. Data are represented as fold change from each fASM line control. *P < 0.05 by unpaired *t* test. In **C** and **D**, data are represented as mean ± SEM; *n* = 5 fASM lines/group.

fASM was treated with GYY4137 or AP39 and exposed to 21% or 40% O_2_ for 48 hours followed by staining with MitoSOX Red (fluorescent mitochondrial superoxide indicator). In vehicle controls, 40% O_2_ induced a marked increase in mitochondrial ROS production, and GYY4137 substantially attenuated this effect (Figure 6A; representative MitoSOX images). AP39 also substantially decreased 40% O_2_–induced mitochondrial ROS (Figure 6B; representative MitoSOX images). Seahorse-based assessment of mitochondrial oxygen consumption rates (OCRs) during mitochondrial stress testing (Figure 6C) showed that GYY4137 increased basal respiration in 21% O_2_, with a marked increase in maximal respiration under the same conditions. GYY4137 blunted the effects of hyperoxia on maximal respiration (Figure 6D).

### H_2_S attenuates O_2_ effects on calcium through the cAMP pathway.

**Figure 7:**
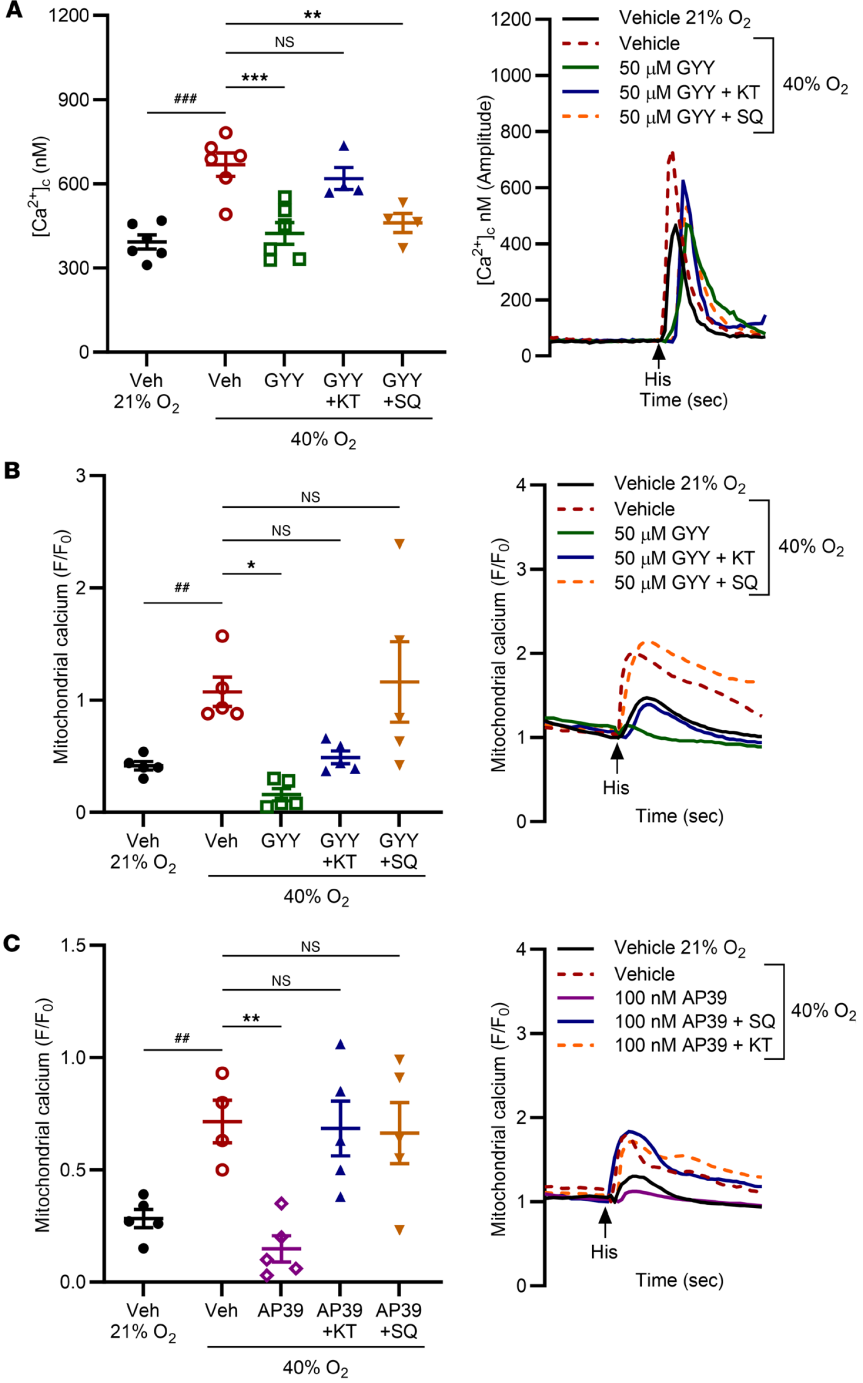
H_2_S attenuates O_2_ effects on calcium through the cAMP pathway. (**A** and **B**) fASM cells were treated as follows: vehicle in 21% O_2_, vehicle in 40% O_2_, 50 µm GYY4137 in 40% O_2_, 50 µm GYY4137 + 100 nM KT5720 (PKA inhibitor) in 40% O_2_, or 50 µm GYY4137 + 10 µm SQ22536 (AC inhibitor) in 40% O_2_. (**A**) Cells were loaded with 5 µm Fura-2/AM for live cell fluorescent imaging of [Ca^2+^]_c_ response to 10 µm histamine. Amplitude [Ca^2+^]_c_ responses were calculated as in Figure 3C. Representative tracings (1 of 6) are shown. (**B**) Cells were loaded with 1 µm Rhod-2 for live cell fluorescent imaging of [Ca^2+^]_m_ response to 10 µm histamine. [Ca^2+^]_m_ response was calculated as in Figure 3D. Representative tracings are shown (1 of 5). (**C**) fASM cells were treated as follows: vehicle in 21% O_2_, vehicle in 40% O_2_, 100 nM AP39 in 40% O_2_, 100 nM AP39 + 100 nM KT5720 in 40% O_2_, or 100 nM AP39 + 10 µm SQ22536 in 40% O_2_. Cells were loaded with 1 µm Rhod-2 for live cell fluorescent imaging of [Ca^2+^]_m_ response to 10 µm histamine. Representative tracings are shown (1 of 5). ^##^*P* < 0.01; ^###^*P* < 0.001 by unpaired *t* test; *P < 0.05; **P < 0.01; ***P < 0.001 by 2-way ANOVA with Bonferroni’s correction for multiple comparisons. Data are represented as mean ± SEM; *n* = 5–6 fASM lines/group.

The alleviating role of cAMP in adult ASM and bronchodilation is well established (40–42), but much less is known about the role of cAMP in the developing airway. To assess the involvement of cAMP in H_2_S-mediated attenuation of [Ca^2+^]_c_ in moderate O_2_, we treated fASM cells with either SQ22536, an adenylate cyclase (AC) inhibitor or KT5720, a PKA inhibitor. Vehicle- (DMSO for SQ22536, methanol for KT5720) or inhibitor-treated fASM cells were exposed to 21% or 40% O_2_ prior to imaging of [Ca^2+^]_c_ responses to histamine. GYY4137 attenuated hyperoxia-induced increases in [Ca^2+^]_c_, an effect reversed by PKA inhibition but not AC inhibition, suggesting partial PKA dependence (Figure 7A; representative images). For [Ca^2+^]_m_ (measured using Rhod-2), both AC and PKA inhibition blunted the GYY4137-mediated attenuating effect in 40% O_2_, indicating cAMP pathway involvement (Figure 7B). Exposure to mitochondria-targeted donor AP39 showed similar results where AC or PKA inhibition restored [Ca^2+^]_m_ responses to hyperoxic control levels (Figure 7C).

### Exogenous H_2_S donors attenuate O_2_ effects on fASM cell contractility.

**Figure 8:**
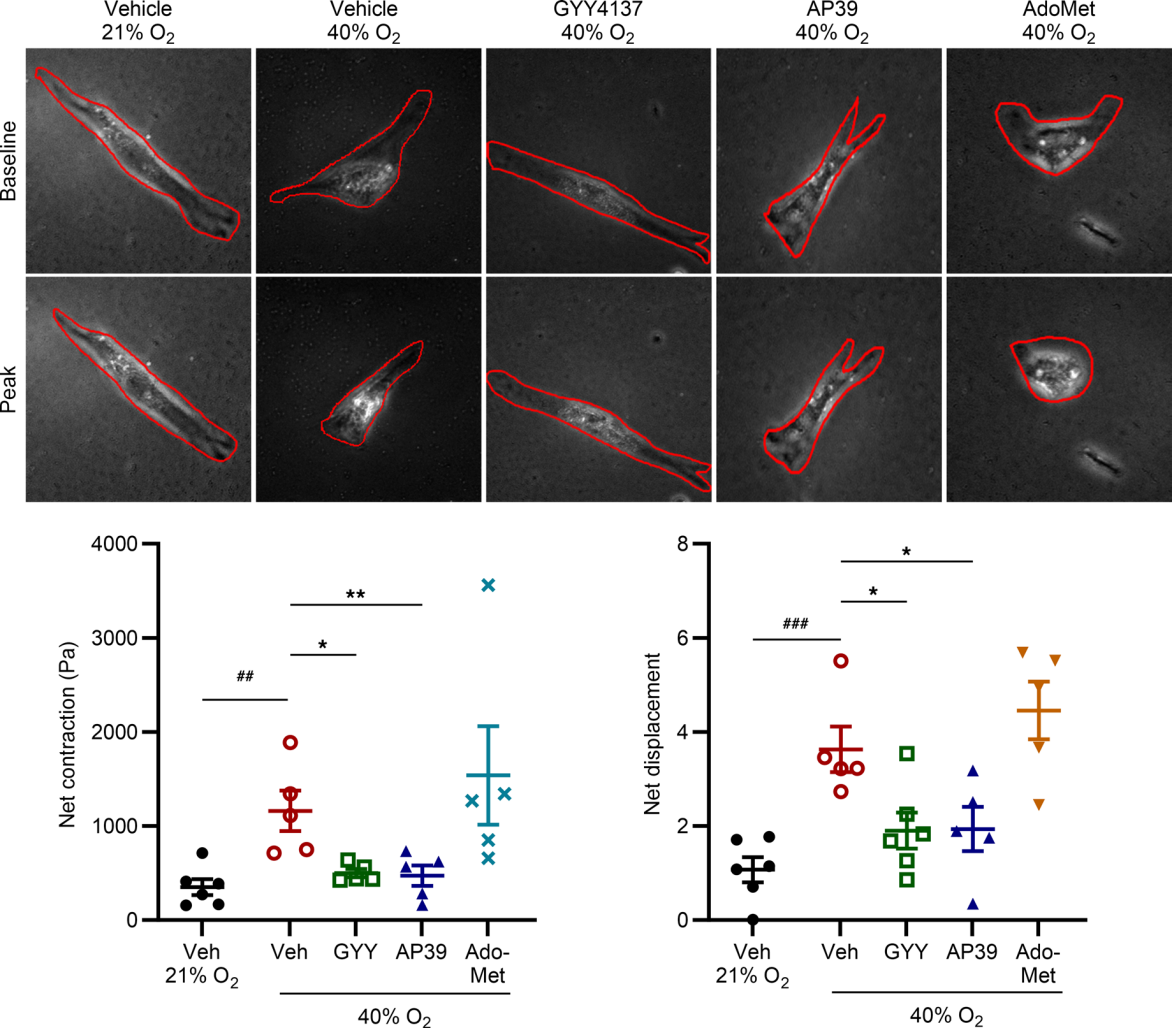
Exogenous H_2_S donors attenuate O_2_ effects on fASM cell contractility. Hydrogels were seeded with fluorescent sulfate–modified latex microspheres and coated with collagen prior to fASM cell plating and subsequent treatment with 50 µm GYY4137, 100 nM AP39, or 1 mM AdoMet and exposure to either 21% or 40% O_2_ for 48 hours. Traction force images were first taken to calculate a baseline. Wells were then injected with 10 µm histamine and images captured at 30-second intervals for 5 minutes on a Cytation 5 imaging system. Two-dimensional tractions were calculated from acquired images by measuring net bead displacement and contraction. ^##^*P* < 0.01; ^###^*P* < 0.001 by unpaired *t* test; *P < 0.05; **P < 0.01 by 2-way ANOVA with Bonferroni’s correction for multiple comparisons. Data are represented as mean ± SEM; *n* = 5–6 fASM lines/group. Representative TFM images are from 1 of 6 fASM lines used in contractility experiments.

Traction force microscopy (TFM) was used to investigate effects of H_2_S on contractility in fASM cells treated with vehicle, GYY4137, AP39, or AdoMet and exposed to 21% or 40% O_2_ for 48 hours. 40% O_2_ increased net contraction and net displacement of fASM cells. Both GYY4137 and AP39 attenuated these effects of hyperoxia on contractility, while AdoMet did not have an attenuating effect (Figure 8).

### H_2_S donors increase p-CREB expression.

**Figure 9:**
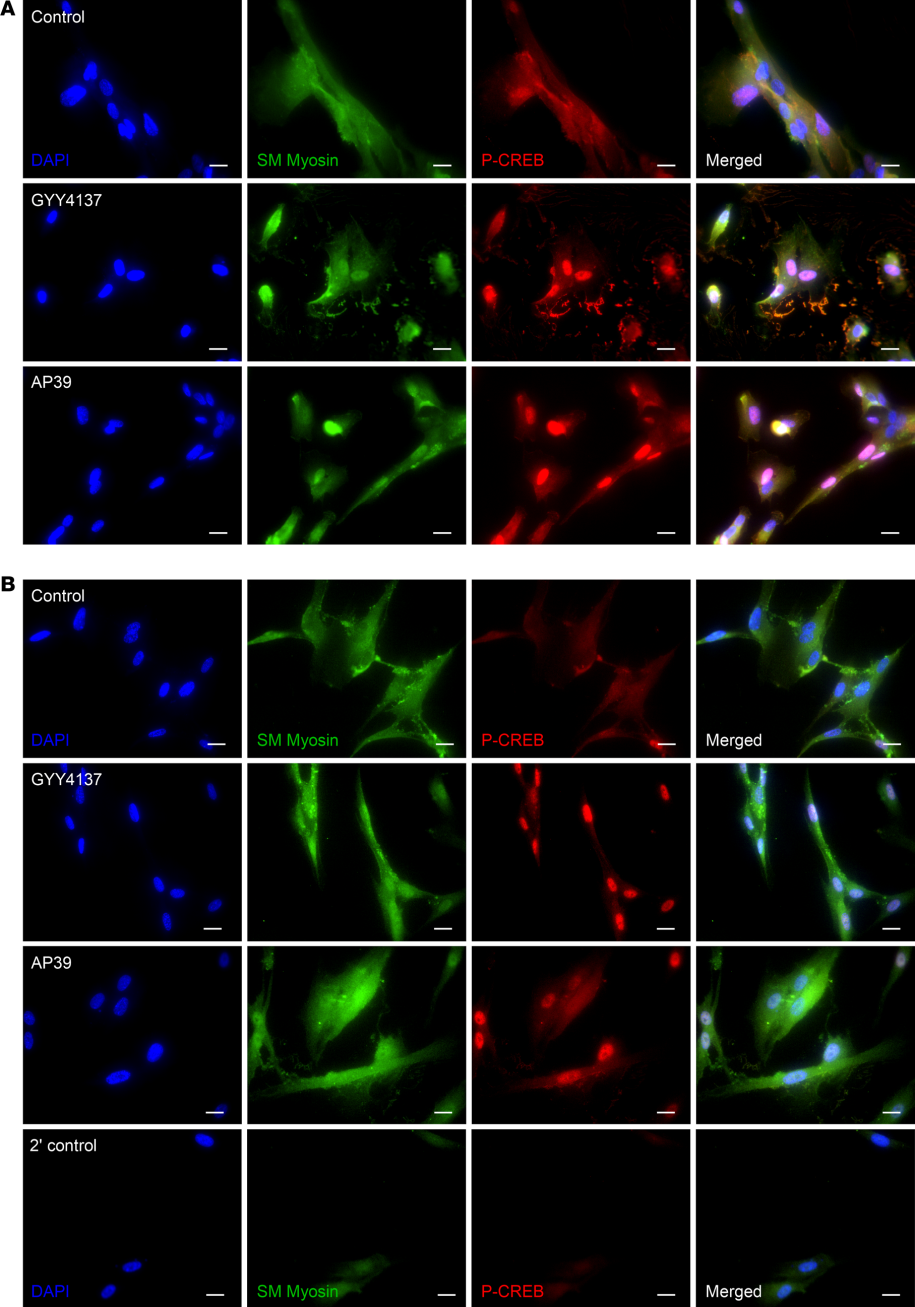
H_2_S donors increase p-CREB expression. Untransduced fASM cells were treated for (**A**) 2 hours or (**B**) 24 hours with vehicle control, 50 µm GYY4137, or 100 nM AP39 prior to exposure to 21% O_2_. fASM cells were fixed, permeabilized, and stained for p-CREB or smooth muscle myosin, and mounted with DAPI. Representative images are 1 of 3 immunofluorescence experiments. Images were captured using a Nikon Eclipse Ti imaging system. Blue, DAPI; green, smooth muscle (SM) myosin; red, p-CREB. Scale bars: 20 μm.

Phosphorylated cAMP response element–binding protein (p-CREB) is a key regulatory downstream target of PKA and an indicator of cAMP pathway activation. We used untransduced fASM cells treated with either GYY4137 or AP39 and exposed to 21% O_2_ to assess p-CREB expression via immunofluorescence. At 2 and 24 hours, GYY4137 or AP39 both increased p-CREB (Figure 9).

## Discussion

Understanding mechanisms by which early hyperoxia induces sustained changes in premature airways leading to subsequent chronic airway disease is vital for improving long-term therapeutic outcomes. H_2_S has emerged as a promising therapeutic candidate, with increasing evidence for its role in lung health and disease (43). However, fewer studies have focused on H_2_S signaling and its downstream effects in premature developing airways. Our foundational studies explored H_2_S in developing human ASM and in a neonatal hyperoxia model, establishing the relevance of endogenous H_2_S systems and the therapeutic potential of exogenous H_2_S donors in the developing lung. We previously showed that human fASM expresses core H_2_S metabolism and functional machinery, and that moderate hyperoxia blunts CBS, H_2_S, and downstream pathways (20). We demonstrated that exogenous H_2_S donors (NaHS, GYY4137) attenuate hyperoxia-induced airway dysfunction in vivo (21). Overall, our studies show that loss of H_2_S is particularly detrimental in hyperoxia, impairing ASM function (20, 21), and underscoring the need to understand targetable mechanisms underlying the alleviating effects of H_2_S.

In the present study, we expanded upon our previous findings to identify mechanisms of H_2_S-mediated protection in moderate hyperoxia. With the hypothesis that exogenous H_2_S counteracts O_2_-induced mitochondrial dysfunction in human fASM, we used stable CBS-KD and control fASM cells exposed to 21% versus 40% O_2_ to investigate mitochondrial structure and function. We also used CBS stabilization via AdoMet versus exogenous H_2_S donors (slow-releasing H_2_S donor GYY4137 and mitochondrial specific H_2_S donor AP39) to investigate cytosolic calcium effects, mitochondrial structure, function, respiration, and cAMP signaling. Our data support a critical role for CBS and demonstrate that GYY4137 and AP39 attenuate O_2_-induced mitochondrial dysfunction in fASM. Our findings therefore suggest that H_2_S counteracts deleterious effects of moderate hyperoxia on mitochondrial homeostasis. Furthermore, we show that O_2_-induced exaggeration of [Ca^2+^]_c_ and [Ca^2+^]_m_ responses can be alleviated through H_2_S modulation via cAMP signaling (PKA or AC).

Although H_2_S holds strong therapeutic promise, dosing of exogenous H_2_S donors remains a challenge. Exogenous H_2_S may be therapeutically beneficial, but excessive levels are cytotoxic (44), making it critical to understand and target H_2_S within a physiological range to achieve maximum therapeutic potential without risking toxicity or safety. We used donor and AdoMet concentrations that align with previous studies and pilot dose-response experiments (data not shown). For example, 1 mM AdoMet saturates both high- and low-affinity AdoMet binding sites on CBS, maximizing allosteric activation and ensuring robust stabilization (38, 45). In the present study, CBS stabilization via AdoMet effectively attenuated O_2_ effects on fASM [Ca^2+^]_c_ and [Ca^2+^]_m_, but did not reverse hyperoxia effects on mitochondrial respiration or contractility (TFM). These unexpected findings highlight the dynamic nature of H_2_S metabolism and the importance of tight control within a physiological range, where prolonged CBS stabilization may increase H_2_S levels beyond a therapeutically relevant range. This idea is supported by the inadvertent negative effects of high H_2_S levels in normoxia where AdoMet decreases mitochondrial respiration in 21% O_2_. Alternatively, hyperoxia may disrupt the transsulfuration pathway to limit the efficacy of CBS stabilization alone.

Given our data with AdoMet and CBS stabilization, exogenous H_2_S donors hold greater potential in the context of hyperoxia. In our studies, we used slow-releasing donors GYY4137 and AP39. The benefit of GYY4137 is that it can interact with multiple cellular compartments, while AP39 targets H_2_S delivery to the mitochondria, with subsequent pronounced mitochondria-specific effects (46, 47). We previously tested multiple doses of GYY4137 in fASM cells in preparation of our in vitro and in vivo studies (20, 21) and found 50 µm GYY4137 to be well within the range of commonly used concentrations in the field (10–400 �m in vitro), and producing physiologically relevant levels of H_2_S without cytotoxicity (48). Similarly, 100 nM AP39 following a pilot dose-response study (data not shown) falls within the effective range (30–100 nM) shown in endothelial cells, neurons, and renal epithelial cells (49–51) without inducing cytotoxicity (250–300 nM). In our previous studies using fASM and our neonatal mouse hyperoxia model, we showed that exogenous H_2_S has a beneficial role specific only under moderate O_2_ exposure (20, 21). Consistent with our prior work, exogenous H_2_S was beneficial only under moderate hyperoxia; manipulation under normoxia may shift H_2_S outside its safe physiological range, leading to adverse effects. Thus, understanding H_2_S mechanisms in hyperoxia is essential for therapeutic translation.

Our data show that H_2_S signaling is complex and context dependent, making it important to understand how best to increase H_2_S and what effects it may have in hyperoxia. General H_2_S donors such as GYY4137 sometimes provided more benefit compared with a mitochondria-specific donor (AP39), while indirect regulation of H_2_S through CBS stabilization was less effective in the context of contractility, for example, as demonstrated by our TFM data. In the context of complexity, it is also important to consider that H_2_S signaling has crosstalk with nitric oxide (NO; discussed below), is involved in protein modification of cysteine residues through persulfidation, and involves other synthetic enzymes not investigated in the present manuscript (3-mercaptopyruvate sulfurtransferase [3MST], cysteine aminotransferase [CAT], and D-amino acid oxidase [DAO]) (43, 52). Furthermore, CBS stabilization affects more than just H_2_S production, and is involved in the transsulfuration pathway, methionine cycle, and GSH production (38, 39, 43, 45, 52). Overall, our data suggest a future focus on testing and validation of exogenous H_2_S donors where differential effects of general donors versus those targeting mitochondria could be leveraged toward alleviating different detrimental effects of hyperoxia on calcium/contractility versus mitochondria and metabolism.

The bronchodilatory effects of H_2_S in developing ASM may be mediated through cAMP signaling. While cAMP-mediated bronchodilation is well established in adults (40–42), its role is less well understood in neonatal airways and fASM (53–56). In adults, bronchodilation can be driven by both cAMP (e.g., β-agonists) and cyclic GMP (cGMP) via NO (57–59). However, NO and cGMP signaling is dysfunctional in developing ASM exposed to O_2_ (60–62), aligning with limited clinical efficacy of inhaled NO in premature infants (61, 63–67). β2-AR agonists that induce bronchodilation in adult asthmatics (68, 69) increase AC activity and cAMP, which activates PKA, eliciting downstream effects such as inhibition of Ca^2+^ influx and accelerated SR Ca^2+^ reuptake (68, 69). We previously showed that cAMP signaling is in fact maintained in fASM even in moderate hyperoxia, with PKA activity unchanged (56). Here, exogenous H_2_S has cell- and context-specific effects on cAMP; studies have shown that NaHS increases cAMP and PKA in neuronal, epithelial, and smooth muscle cells promoting dilation, but H_2_S can blunt β2-AR effects in vasculature (31, 32). Regardless, if H_2_S has bronchodilatory effects in developing ASM, then elevation of cAMP would be beneficial particularly in hyperoxia where cAMP signaling is retained. Accordingly, exploring targetable mechanisms of bronchodilation through cAMP pathways offers promising therapeutic potential, and H_2_S may be a key component in this effort.

Our studies also reveal the differential effects of 2 slow-releasing H_2_S donors, GYY4137 and AP39. Mitochondria-targeted AP39 increases mitochondrial calcium retention capacity, and decreases mitochondrial permeability transition pore opening, limiting activation of cell death pathways (70). GYY4137 may exert its effects on calcium handling differently. In human umbilical vein endothelial cells, GYY4137 increases mitochondrial electron transport and oxygen consumption via sulfide:quinone oxidoreductase (SQOR), a key enzyme for H_2_S metabolism (71). GYY4137 can also induce posttranslational modification by H_2_S. For example, persulfidation by H_2_S affects protein activity, localization, and protein-protein interaction, all of which impact on cellular and mitochondrial function (43, 71–73). Because of these key differences between GYY4137 and AP39, it is reasonable that our mechanistic data involving KT55720 (PKA inhibitor) and SQ22536 (AC inhibitor) would yield differential results; only PKA inhibition blocked the attenuating effect of GYY4137 on [Ca^2+^]_c_ in 40% O_2_, while both PKA inhibition and SQ inhibition blocked the attenuating effect of AP39 on [Ca^2+^]_m_ (PKA to a lesser extent). Thus, it is plausible that the effects of cAMP pathway modulation have differential effects on [Ca^2+^]_c_ versus [Ca^2+^]_m_, with the expectation that [Ca^2+^]_m_ effects would be more pronounced with AP39 treatment and its influence on cAMP.

Therapeutic use of H_2_S and its downstream signaling are especially intriguing for premature infants given their differential response to cAMP versus cGMP modulation compared with adults. For example, NO is known to reduce airway tone, proliferation, and remodeling in adults (57–59) but inhaled NO is ineffective in preventing airway disease during the perinatal period (61, 63–67). In adult airways, the NO/soluble guanylyl cyclase (sGC)/cGMP axis can induce bronchodilation but in neonates, dysfunctional sGC is thought to blunt NO effects on bronchodilation (74). Thus, bypassing dysfunctional sGC may be needed in premature infants, and H_2_S may be the agent to accomplish this goal. Studies have shown that H_2_S can regulate the sGC redox state that is key to sGC function and responsiveness to NO (75, 76). Others have shown H_2_S enhances NO production and downstream signaling effects in the vasculature (75, 76), potentially though H_2_S stimulation of eNOS synthesis and activity (77–79). Separately, we showed that increased cGMP production via sGC activation can also alleviate O_2_-induced effects on [Ca^2+^]_c_ responses to bronchoconstrictors (60). It is thus plausible that in addition to beneficial effects on the cAMP pathway, exogenous H_2_S could be therapeutically beneficial by enhancing sGC-NO responsiveness in premature airways in hyperoxia, which is an appealing area for further exploration.

Another beneficial aspect of H_2_S may be in the context of antioxidant regulation. This is particularly relevant in premature infants given that antioxidant systems (e.g., catalase, GSH peroxidase, superoxide dismutase), which mature later in gestation in the fetal lung for a normal postnatal transition (9–11), are ill-prepared for early shift from in utero hypoxia to ambient normoxia. Unfortunately, antioxidant therapies (e.g., *N*-acetylcysteine) have not shown clinical efficacy. In this scenario, H_2_S presents an opportunity to leverage readily available exogenous H_2_S donors to target mitochondrial structure/function and cellular redox status (28–30). For example, the antioxidant GSH is critically linked to H_2_S signaling in mitochondria. H_2_S helps redistribute GSH to the mitochondria, driving cytoprotection (80), and increases overall GSH production, e.g., in neurons (26). Our data in this study showing reduction of ROS by H_2_S donors point to H_2_S as a compelling candidate for future studies targeting oxidative stress in the developing lung exposed to moderate O_2_.

While the current study focused on H_2_S and mitochondria, calcium and cAMP, it is important to acknowledge other mechanisms also relevant to developing airways. H_2_S donors, including GYY4137 and AP39, directly inhibit phosphodiesterases (PDEs), leading to increased cAMP or cGMP (81–83). Thus, the increased cAMP in our studies may also result from inhibited PDEs. Furthermore, CBS removes homocysteine through conversion to cystathionine. With dysfunctional or reduced CBS levels, accumulation of homocysteine could potentially lead to increased ROS, mitochondrial dysfunction, oxidative stress, and altered calcium homeostasis, the latter involving the extracellular calcium–sensing receptor (CaSR) (84), a factor we previously showed to be a key mediator of hyperoxia effects in fASM (36). Separately, H_2_S-mediated persulfidation, which is the addition of a sulfur molecule onto a reactive thiol group of a cysteine residue (R-SH to R-SSH), is a redox mechanism that regulates diverse aspects of H_2_S signaling, acting as a “switch” for protein function and subcellular localization (72, 73, 85, 86). Under cellular stress, H_2_S could increase protein persulfidation, influencing mitochondrial homeostasis, intracellular calcium, and overall cellular function (72, 86), aspects that remain unexplored in the context of developing ASM or hyperoxia-induced cell stress.

In summary, exogenous H_2_S donors more so than CBS stabilization attenuate moderate hyperoxia–induced mitochondrial dysfunction in developing ASM, partly via cAMP signaling. Targeting premature airways during hyperoxia exposure is critical to preventing AHR and remodeling. Our findings highlight H_2_S as a promising therapeutic strategy for protecting underdeveloped lungs exposed to moderate hyperoxia.

## Methods

### Sex as a biological variable.

Our study examined male and female fASM cells, and findings are reported collectively.

### Human fASM.

Human fetal ASM (fASM) was enzymatically dissociated from tracheobronchial trees following fetal demise at 18–22 weeks of gestation, as previously described (37, 87). fASM cells are de-identified, considered exempt by the Mayo Institutional Review Board, and not considered Human Subjects Research. Characteristics of fASM cells (expression of smooth muscle markers and factors involved in calcium regulation) are published (37, 88, 89).

### Cell culturing and hyperoxia exposure.

fASM cells cultured (< passage 8) in Dulbecco’s modified Eagle’s medium (DMEM)/F12 (Life Technologies) supplemented with 10% FBS and 1% penicillin-streptomycin (Life Technologies). fASM cells were incubated in either 21% O_2_/5% CO_2_ or 40%–50% O_2_/5% CO_2_ humidified incubators for experiments. After 24 hours in respective O_2_ conditions, fASM cells were serum deprived in medium containing 0.5% FBS and placed in respective O_2_ incubators for another 24 hours.

### Compounds and treatments.

Exogenous H_2_S donor GYY4137 (slow-releasing/“chronic”) was purchased from Tocris Bio-Techne (catalog 3658). Mitochondrial H_2_S exogenous donor AP39 was purchased from Cayman Chemical (catalog 17100). CBS activator AdoMet was purchased from Sigma-Aldrich (catalog A7007). AC inhibitor SQ22536 was purchased from Tocris Bio-Techne (catalog 1435). PKA inhibitor KT5720 was purchased from Sigma-Aldrich (catalog K3761).

### shRNA lentiviral lines.

Human fASM lines were used to generate CBS-KD lines by infection with CBS shRNA Lentiviral Particles (Santa Cruz Biotechnology, sc-60335-V) and Control shRNA Lentiviral Particles-A (Santa Cruz Biotechnology, sc-108080). After fASM cells reached approximately 60% confluence, DMEM/F12 was added to the cells with 5 mg/mL Polybrene (Santa Cruz Biotechnology, sc-134220). shRNA lentiviral particles were added to the cells and incubated overnight. A minimum of 3 different volumes of lentiviral particles were used to determine optimal transduction efficiency. After 24 hours, medium was replaced with complete fASM growth medium. Following another 24-hour incubation, selection for stable clones began using 5 mg/mL puromycin dihydrochloride, determined empirically (Gibco/Thermo Fisher Scientific, A1113803). fASM cells were refreshed with puromycin-containing media for at least 4 days, until resistant cells could be identified. Every transduction included a mock transduction to ensure cell death following the addition of puromycin-containing media.

### Mitochondrial morphology and mitochondrial ROS.

Human fASM cells treated with exogenous H_2_S donors and exposed to 21% or 40% O_2_, or CBS-KD/Scr shRNA fASM cells exposed to 21% or 40% O_2_ were subject to mitochondrial analysis. Cells were dyed with 400 nM MitoTracker Green FM (ex/em 490/516 nm; Invitrogen/Thermo Fisher Scientific, M7514) and 2 �m MitoSOX Red (ex/em 396/610 nm; Invitrogen/Thermo Fisher Scientific, M3008) for 30 minutes. Negative controls (MitoTracker only) were used for imaging. After washing off dye, an inverted fluorescence microscopy (Keyence BZ-X800E) was used to image the cells. ImageJ software from the NIH (90) and MitoMorph, a publicly accessible ImageJ macro, were used to measure single cells and mitochondrial morphology following correction for background fluorescence. Mitochondrial networks were analyzed for area, perimeter, circularity, and major/minor axes. Form factor (mitochondrial branching/connections was calculated in MitoMorph as the following: form factor = perimeter^2^/(4π × area). Aspect ratio (mitochondrial branch length) was calculated in MitoMorph as the following: aspect ratio = ratio of major to minor axes of the ellipse equivalent to the object (35, 91).

### Immunofluorescence.

The following groups were used for immunofluorescence: CBS-KD or Scr shRNA fASM cells exposed to 21% or 40% O_2_, and untransduced fASM cells treated with 2 hours or 24 hours GYY4137 (50 mM) or AP39 (100 nM) in 21% O_2_. In all groups, fASM cells were fixed with 4% paraformaldehyde for 10 minutes, washed 3 times with Tris-buffered saline (TBS), permeabilized for 5 minutes using 0.1% Triton X-100 in TBS, and washed an additional 3 times in TBS. Cells were blocked with 4% donkey serum for 1 hour. For CBS detection, fASM cells were incubated 24 hours at 4°C with 5 mg/mL anti-CBS rabbit antibody (Abcam, ab96252), washed 3 times in TBS, and incubated with 4 mg/mL donkey anti-rabbit–Alexa Flour 647 (Invitrogen, A31573) secondary antibody for 1 hour at room temperature. For p-CREB detection, fASM cells were incubated 24 hours at 4°C with 2.5 mg/mL rabbit anti–p-CREB antibody (Cell Signaling Technologies, 9198) and mouse anti–smooth muscle myosin (Sigma-Aldrich, M7786; 1:500). fASM cells were washed 3 times with TBS and incubated with 10 mg/mL donkey anti-rabbit–Alexa Fluor 555 (Invitrogen, A31572) and 4 mg/mL donkey anti-mouse–Alexa Fluor 488 (Invitrogen, A21202) secondary antibodies for 1 hour at room temperature. Following secondary antibody incubation, all fASM cells were rinsed 3 times in TBS and coverslips mounted using FluoroGel II with DAPI (Electron Microscopy Sciences, 17985-50). Fluorescence was detected using standard filter sets and a Nikon Eclipse Ti imaging system.

### Cytoplasmic and mitochondrial calcium imaging.

Real-time fluorescence imaging of cytoplasmic and mitochondrial calcium followed established techniques previously published (20, 37, 56, 92). Briefly, fASM cells were plated in 8-well chambered coverslips (Ibidi) and exposed to 21% or 40% O_2_. For cytoplasmic calcium imaging, fASM cells were loaded with 5 µm Fura-2/AM (Thermo Fisher Scientific), a ratiometric cytoplasmic calcium indicator dye, in 1× HBSS (containing 2 mM CaCl_2_, 1 mM MgSO_4_, HEPES, pH 7.4) for 30 minutes. Dye was washed off the cells with HBSS prior to imaging. Live-cell imaging was performed using an inverted microscope (Nikon Eclipse Ti-U) using filters to detect Fura-2/AM (Ex/Em: 340 nm and 380/410 nm). Following continuous perfusion with 1× HBSS, 10 µm histamine was perfused and measurements recorded for baseline, peak, and amplitude of cytoplasmic calcium responses. 10–15 cells/well were measured.

For mitochondrial calcium imaging, fASM cells were loaded with 1 µm Rhod-2/A (Thermo Fisher Scientific), a mitochondrial calcium indicator dye, in 1× HBSS for 45 minutes at room temperature. Dye was washed off the cells with HBSS prior to live-cell imaging on an inverted microscope (Nikon Eclipse Ti imaging system, LED fluorescence light source, 16-bit high-sensitivity CCD camera) using filters to detect Rhod-2/A (Ex: 555 nm, Em: 580 nm using a 560/55 nm bandpass filter at 1 Hz, 200 ms exposure), as previously described (93). fASM cells were continuously perfused with 1× HBSS while recording measurements. During recordings, baseline fluorescence was first established (~30–60 seconds) followed by perfusion with 10 µm histamine until response was visualized. Ten to 15 cells/well were measured, with 2 ROIs placed per cell. Three to 4 ROIs were placed outside of cells to determine background measurements for analysis.

### Mitochondrial stress tests.

Human fASM cells were subjected to mitochondrial stress tests using a Seahorse XFe Bioanalyzer (Agilent Technologies). Cells were plated in Agilent Seahorse XF24 Cell Culture Microplates (Agilent, 100777-004) at a density of 2 × 10^4^ cells/well and exposed to either 21% or 40% O_2_ for 24–48 hours (based on rate of each line’s cell growth), with or without exogenous H_2_S donors GYY4137 or AP39. XF Base Medium (Agilent Technologies, 103334-100) was supplemented with 10 mM glucose, 1 mM sodium pyruvate, and 2 mM glutamine at pH 7.4 for assays. Twenty-four hours prior to assays, Seahorse XFe24 FluxPak sensor cartridges were hydrated. Concentrations of mitochondrial stress test reagents were experimentally validated for fASM cells and based on manufacturer guidelines. Final concentrations were as follows: 1 μM oligomycin (Sigma-Aldrich, 75351), 1.25 μM FCCP (Sigma-Aldrich, c2920), 1 μM antimycin A (Sigma-Aldrich, A8674), and 1 μM rotenone (Sigma-Aldrich, R8875). The Seahorse Bioanalyzer protocol was set to mix (1 minute), wait (2 minutes), and measure (3 time points) after addition of each compound. Values were normalized by in situ cell counts using 1 mg/mL Hoechst 33342 Solution (Thermo Fisher Scientific, 62249) and imaging on a Cytation 5 plate reader (BioTek/Agilent). OCR measurements were obtained prior to addition of reagents and following sequential addition of oligomycin (inhibits ATP synthase), FCCP (proton gradient uncoupler), and rotenone (complex I inhibitor) plus antimycin A (complex III inhibitor). Basal respiration and maximal respiration were calculated based on difference between measurement points 1–3 and 10–12 (basal) or measurement points 7–9 and 10–12 (maximal).

### Protein expression.

Protein was isolated using RIPA lysis buffer (Thermo Fisher Scientific, 89901) and 1× Protease and Phosphatase Inhibitor Cocktail (Thermo Fisher Scientific, 1861280) following the manufacturer’s protocol. Protein was quantified using DC Protein Assay kit (Bio-Rad, 5000111) and FlexStation plate reader. A Jess Automated Western Blot System by Protein Simple (ProteinSimple, Bio-Techne Brand, 004–650) was used to measure protein expression. This system is an automated and quantitative digital Western blot technology that uses a capillary immunoassay system for protein quantification. Approximately 0.3 mg protein was loaded per capillary and appropriate antibody concentration was determined. Primary antibodies used were anti-CBS (D8F2P) rabbit mAb (Cell Signaling Technologies, 14782; 1:25) and anti-CTH rabbit pAb (Abcam, ab136604; 1:50). CBS and CSE protein expression values were normalized to total protein via the Total Protein Detection Module for Chemiluminescence (ProteinSimple, Bio-Techne Brand, DM-TP01), following the manufacturer’s protocol. Anti-Rabbit Secondary HRP Antibody (ProteinSimple, Bio-Techne Brand, rabbit, 042-206) is supplied at a predetermined concentration suitable for Jess Technology and as part of the Anti-Rabbit Detection Module (ProteinSimple, Bio-Techne Brand, rabbit, DM-001).

### TFM.

fASM cell contractility was measured using established TFM techniques (94), as previously described (36, 95, 96). In brief, hydrogels (25 kPa, Matrigen, EasyCoat SoftWell, SW12-EC-25-SYO.2YG) were seeded with fluorescent sulfate–modified latex microspheres (0.2 μm, 505/515 excitation/emission, FluoSpheres, Life Technologies, F8848) suspended in PBS for 30 minutes and then aspirated. Hydrogels were then coated with 0.05 mg/mL type I rat tendon collagen (Alphabioregen, TY005) for 30 minutes and gels washed twice with PBS. fASM cells were plated onto gels (5,000/gel) for 24 hours, then treated with GYY4137, AP39, or AdoMet for 24 hours followed by exposure to either 21% or 40% O_2_ for another 48 hours. Traction force measurement images of gel surface–conjugated fluorescent beads and single cells (phase contrast) were acquired to establish the baseline, wells injected with histamine (10 µm final) and images captured at 30-second intervals for 5 minutes using a Cytation 5 imaging system (10× UPLFLN, NA 0.30 objective; Olympus 10X2PH). Two-dimensional tractions were calculated from acquired images by measuring net bead displacement and contraction using TractionsForAll software, a freely available program from MATLAB (94).

### Statistics.

Prism 10.1.2 software (GraphPad Software) was used for data analysis and figure preparation. Statistical analyses used for each graph are detailed in figure legends. Unpaired 2-tailed *t* tests were used to compare differences between 2 groups, while 2-way ANOVA with Bonferroni’s correction for multiple comparisons was used for assessing more than 2 groups. Outliers were determined by calculating values outside of the average ± 2 × SD. The *n* values represent the number of fASM lines per treatment/exposure group. Data are represented as mean ± SEM and a *P* value of less than 0.05 was used for statistical significance.

### Study approval.

Human fASM cells used in the present studies are de-identified, considered exempt by Mayo Institutional Review Board, and not considered Human Subjects Research.

### Data availability.

Values for all data points in graphs are reported in the Supporting Data Values file.

## Author contributions

CMB, MAT, NAB, MS, YN, YSP, and CP conceived and designed research. CMB, MAT, SKH, NAB, DPK, PR, MS, YN, and CVR performed experiments. CMB and MAT analyzed data. CMB, MAT, SKH, NAB, DPK, PR, MS, YN, CVR, LD, YSP, and CP interpreted results of experiments. CMB and MAT prepared figures. CMB drafted the manuscript. CMB, MAT, SKH, NAB, DPK, PR, MS, YN, CVR, LD, YSP, and CP edited and revised the manuscript. CMB, MAT, SKH, NAB, DPK, PR, MS, YN, CVR, LD, YSP, and CP approved the final version of the manuscript. Co–first authors are listed alphabetically.

## Conflict of interest

The authors have declared that no conflict of interest exists.

## Funding support

This work is the result of NIH funding, in whole or in part, and is subject to the NIH Public Access Policy. Through acceptance of this federal funding, the NIH has been given a right to make the work publicly available in PubMed Central.

- NIH NHLBI T32 HL 105355 (to CMB).
- American Heart Association Postdoctoral Fellowship 20POST35210002 (to CMB).
- NIH NHLBI R01 HL 160570, R01 HL 171915, and R01 HL 177837 (to CP).
- NIH NHLBI R01 HL 158532 and R01 HL 056470 (to YSP).

## Supplementary Material

Unedited blot and gel images

Supporting data values

## Version 1 03/10/2026

In-Press Preview

## Version 2 04/22/2026

Electronic publication
