# Supplementary material for: Hydrogen sulfide alleviates hyperoxia effects on mitochondria in human developing airway smooth muscle
Source: JCI Insight. 2026 Mar 10;11(8):e191475. doi: 10.1172/jci.insight.191475 (PMC13135401; doi:10.1172/jci.insight.191475)
Supplement: Unedited blot and gel images [file jciinsight-11-191475-s143.pdf]

Full unedited gel for Figure 1A

Jess automated Western blot system (Protein Simple) is an automated digital Western blot technology that uses a capillary immunoassay system for protein quantification

Yellow arrow = band quantified

Anti-CBS (D8F2P) Rabbit mAb (Cell Signaling Technologies #14782)
Concentration: 1:25

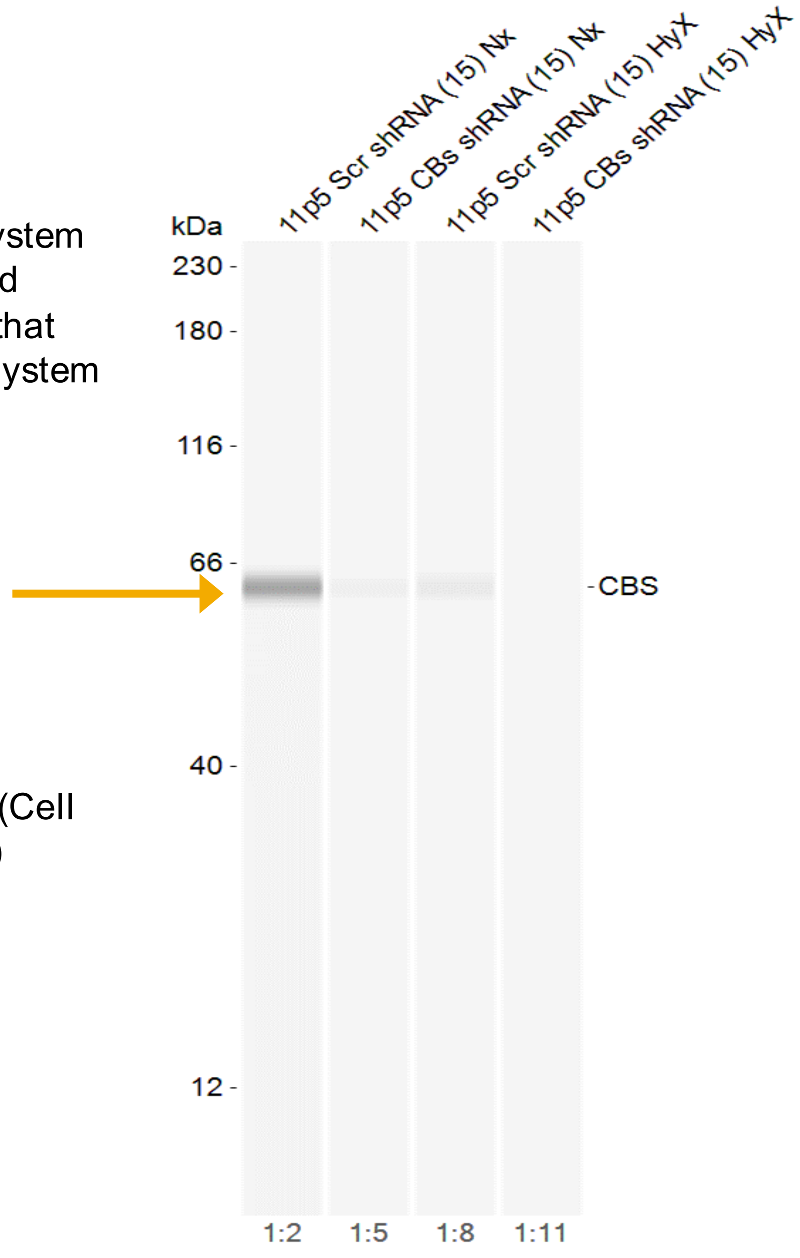

©2026 Mayo Foundation for Medical Education and Research |

Full unedited gel for Figure 1B

Jess automated Western blot system (Protein Simple) is an automated digital Western blot technology that uses a capillary immunoassay system for protein quantification

Anti-Cystathionase/CTH rabbit pAb antibody (Abcam, ab136604 Concentration: 1:50

Yellow arrow = band quantified

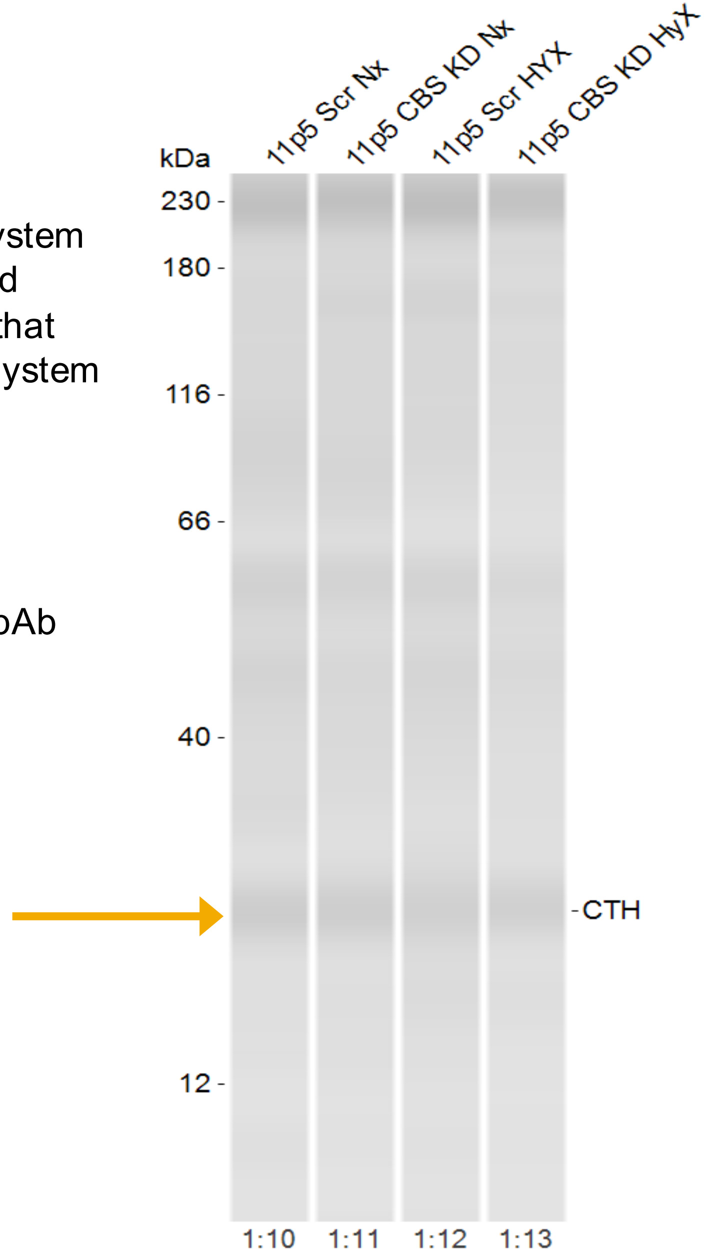

©2026 Mayo Foundation for Medical Education and Research |
